# Human Multipotent Stromal Cells (MSCs) Increase Neurogenesis and Decrease Atrophy of the Striatum in a Transgenic Mouse Model for Huntington's Disease

**DOI:** 10.1371/journal.pone.0009347

**Published:** 2010-02-22

**Authors:** Brooke R. Snyder, Andrew M. Chiu, Darwin J. Prockop, Anthony W. S. Chan

**Affiliations:** 1 Yerkes National Primate Research Center, Atlanta, Georgia, United States of America; 2 Department of Human Genetics, Emory University School of Medicine, Atlanta, Georgia, United States of America; 3 Center for Gene Therapy, Tulane University Health Science Center, New Orleans, Louisiana, United States of America; Case Western Reserve University, United States of America

## Abstract

**Background:**

Implantation of human multipotent stromal cells from bone marrow (hMSCs) into the dentate gyrus of the hippocampus of mice was previously shown to stimulate proliferation, migration and neural differentiation of endogenous neural stem cells. We hypothesized that hMSCs would be beneficial in a mouse model of Huntington disease (HD) due to these neurogenic effects.

**Results:**

We implanted hMSCs into the striatum of transgenic mice (N171-82Q) that are a model for HD. The implanted hMSCs rapidly disappeared over 3 to 15 days. However, they increased proliferation and neural differentiation of endogenous neural stem cells for up to 30 days. They also increased neurotrophic signaling and decreased atrophy of the striatum in 3-month old HD mice implanted with hMSCs one month earlier.

**Conclusions:**

The results therefore suggested that neural implantation of hMSCs may be of benefit in HD but a number of parameters of dose, treatment schedule, and route of administration need to be optimized.

## Introduction

HD is an autosomal dominant genetic disorder caused by the expansion of polyglutamine expeats in Exon 1 of the *IT15* gene encoding for Huntingtin (Htt). The polyglutamine repeat length determines the age of onset and the overall level of function, but not the severity of the disease [1]. Although the exact mechanism underlying HD disease progression remains uncertain, the hallmark of this disease is gross atrophy of the striatum and cortex [2]. The average age of onset for HD is 37–38 years [3] and the median life expectancy after disease onset is 16.2 years (with a span of 2–45 years). The disease duration is similar regardless of age of onset [4]. HD is an extremely debilitating disease, affecting motor, cognition, and psychological stability [5]. According to the 2007 World Congress on HD, there are several clinical trials for treatments for HD, but there is currently no effective therapy (http://www.hda.org.uk/congress/index.html).

One strategy for therapy of HD is to enhance neurogenesis. Neurogenesis is not negatively affected by mutant Htt; in fact, the number of proliferating progenitor cells and new neurons in the subventricular zone (SVZ) and surrounding area was increased in HD patients compared to control [6], [7], [8], [9]. Similarly in the HD rat model created by quinolinic acid injection, neostriatal neurogenesis was increased and neuroblasts migrated to the site of the lesion in the striatum instead of to the olfactory bulb [10]. A transgenic mouse model for HD also suggested an upregulation of neurogenesis, but there was no improvement in the HD phenotype [11]. This natural upregulation of neurogenesis may be further augmented by administration of fibroblast growth factor (FGF-2) [12], [13], nerve growth factor (NGF) [14], [15], and ciliary neurotrophic factor (CNTF) [16], thus improving the pathology and/or phenotype of the disease.

One potential means of increasing neurogenesis in HD is the administration of stem/progenitor cells. Among the stem/progenitor cells currently being tested are the adult stem/progenitor cells from bone marrow referred to initially as fibroblastic colony forming units, then as marrow stromal cells, subsequently as mesenchymal stem cells, and most recently as multipotent mesenchymal stromal cells, or MSCs [17], [18]. MSCs are readily obtained from small samples of bone marrow and expanded in culture. They initially attracted interest as therapeutic agents because they differentiate into multiple phenotypes in culture and in vivo [19], [20]. Initial observations indicated that MSCs infused into the lateral ventricles of newborn or fetal rodents engrafted and probably differentiated into neural cells [21], [22], [23]. Subsequent observations, particularly involving adult animals, indicated that differentiation into neural cells was unlikely to be a major factor [24], [25], [26]. Cell fusion and trans-differentiation may be possible; however, it is unlikely that these completely account for the therapeutic effects accounted to MSCs [25]. Instead, the cells promote repair of the CNS by creating a more favorable environment for neuroprotection or regeneration through the secretion of cytokines and chemokines or other mechanisms [27], [28], [29]. In the immunodeficient adult mice, injection of hMSCs into the dentate gyrus of the hippocampus increased propagation, migration and neural differentiation of endogenous neural stem cells [30]. Also, injection of hMSCs into the dentate gyrus of immunocompetent mice one day after transient global ischemia improved neurological deficits and decreased neuronal death by the modulating immune and inflammatory responses of the mouse brain [31]. In this study, we tested the hypothesis that implantation of hMSCs into the striatum may have beneficial effects in a transgenic mouse model for HD. The cells increased neurogenesis and decreased atrophy of the striatum.

## Results

### Grafted hMSCs Do Not Survive in the HD Striatum

**Figure 1:**
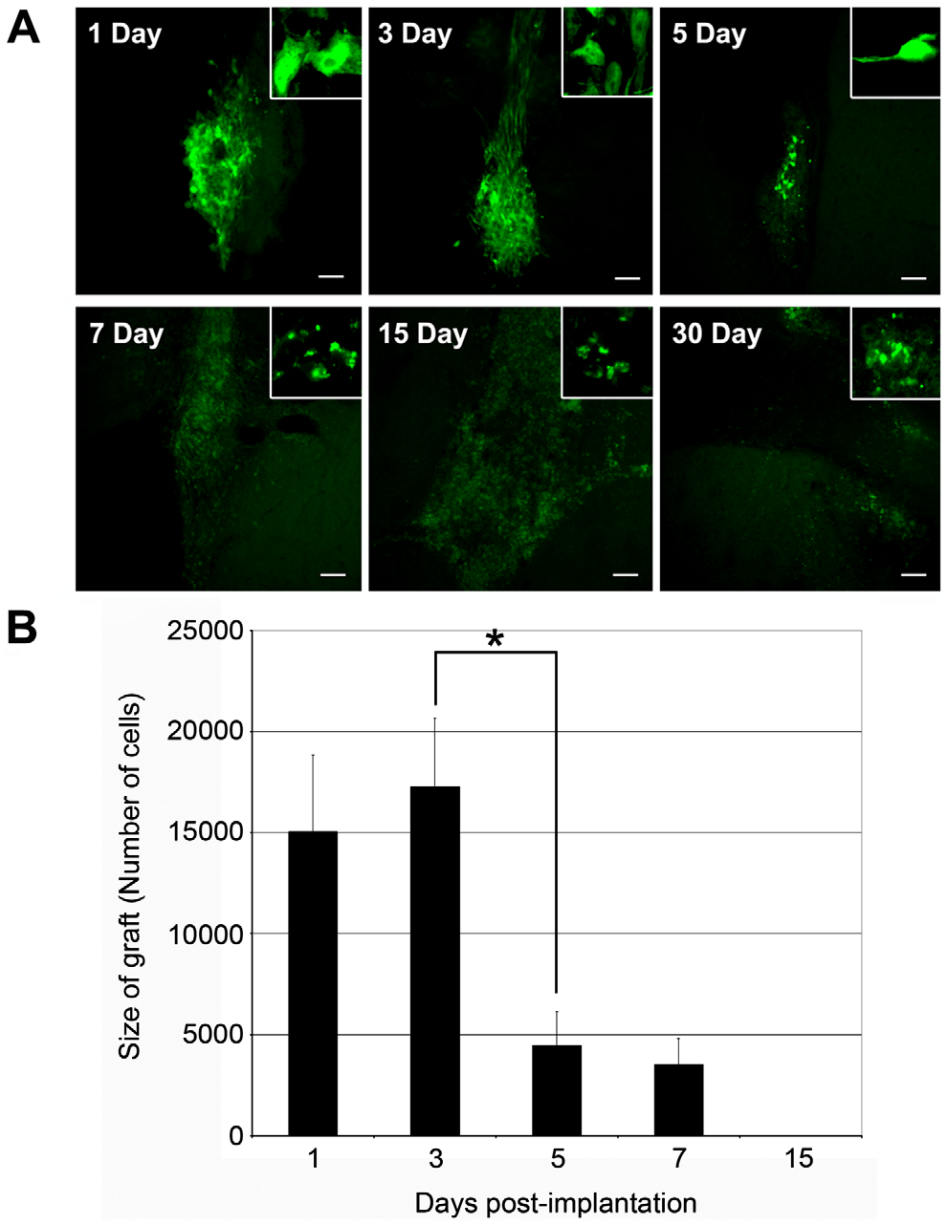
GFP-hMSCs did not survive in the HD striatum. (A) An intact graft of GFP-hMSCs was evident at 1–3 days post-implantation. Only a few intact cells survived to 5 days and only cell remnants remained for 7–30 days. Squares demarcate location of high magnification inset. Scale bar = 100 µm. (B) Stereological cell counting confirmed only 15.1% of the 100,000 grafted hMSCs survived to 1 day and 17.3% to 3 days. Survival significantly dropped (n = 5−6; p = 0.004) to 4.5 and 3.5% at 5 and 7 days. By 15 days no intact cells were counted. * denotes significance based on ANOVA and Holms-Sidak as the post-hoc test.

Previous studies demonstrated that hMSCs implanted into the dentate gyrus disappeared over 5 to 7 days in both immunodeficient and immunocompetent mice [30], [31]. To determine whether implantation of hMSCs would have a similar effect in a mouse model for HD, 100,000 hMSCs were implanted into the striatum of 8 wk old mice (HD N171-82Q) and the brains were examined after 1, 3, 5, 7, 15, and 30 days (n = 5−6). Microscopy for the GFP cells (Figure 1A) at 1 day post-implantation showed that the MSCs had engrafted. Most of these cells survived for at least 3 days post-implantation as cells which retained the spindle-shaped morphology of MSCs (Figure 1A, inset images). By 5 to 7 days post-surgery, the size of the graft was smaller and fewer GFP-hMSCs were present. By 15 days post-implantation, only remnants of the GFP-hMSCs, and no intact cells, were found (Figure S1).

The total number of grafted GFP-hMSCs was also estimated using stereological counting (Figure 1B) on the same sections used for the immunostaining. Only intact hMSCs with typical spindle-shaped morphology were included in this analysis (Figure 1A, inset images). Of the 100,000 cells that were implanted in the striatum, only 15.1% survived the first 24 hours post-operatively (15,063±3,776 cells). There was no significant change at 3 days, with approximately 17.3% of the grafted cells remaining (17,293±3374 cells, p = 0.573). The number of surviving GFP-hMSCs was significantly reduced between 3–5 days post-transplantation, with only 4.5% of the cells remaining (4,481±1678 cells, p = 0.004) and about the same number at day 7 (Not Significant change). At 15 and 30 days post-implantation, intact GFP-hMSCs were not detected.

### Grafting hMSCs into the HD Striatum Significantly Increases Endogenous Cell Proliferation

**Figure 2:**
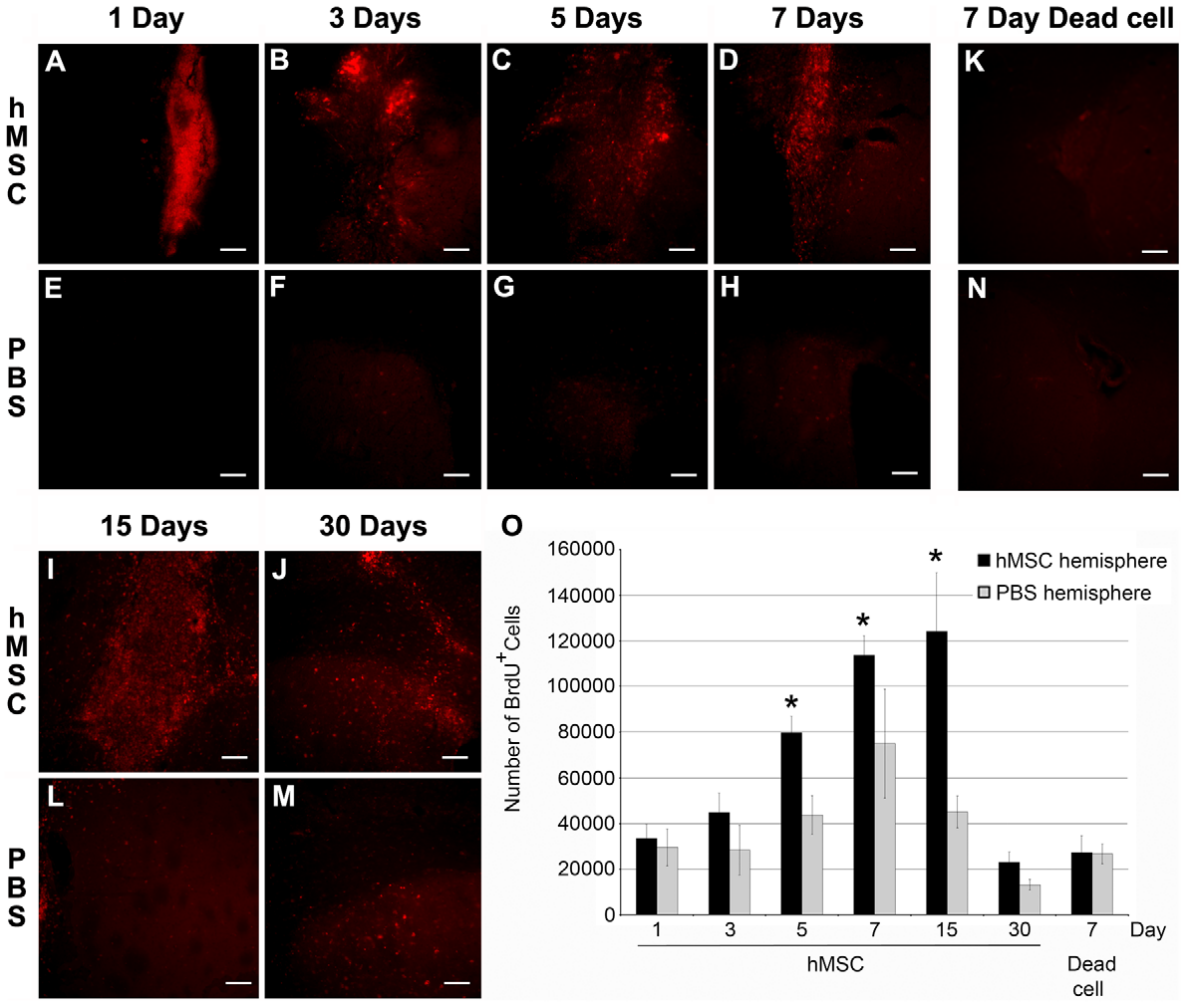
Grafting hMSCs increased the proliferation of endogenous cells in the SVZ and striatum. hMSC implantation increased endogenous cell proliferation at 1-30 days post-implantation (A-D, I-J), but not in the contralateral, PBS-injected hemisphere (E-H, L-M). There was no increase in cell proliferation after dead hMSCs were implanted into the striatum (K,N). (O) hMSCs increased cell proliferation in the striatum from 1 to 15 days post-transplantation (n = 5−6). There were significantly more dividing, BrdU^+^ cells in the hMSC-implanted hemisphere than the PBS-injected hemisphere at 5–15 days post-implantation. There was no difference in the number of BrdU^+^ cells in either hemisphere when dead hMSCs were implanted. * and a denote p<0.05 by ANOVA with Holms-Sidak post-hoc analysis.

Previous observations indicated that grafting hMSCs into the hippocampus of healthy mice increased the proliferation of endogenous neural cells [30]. To determine whether hMSCs enhanced the proliferation of endogenous cells in an HD mouse model, the number of proliferating cells in the SVZ and striatum was assessed by injecting mice with BrdU every 12 hours for the first 7 days post-implantation, thus labeling all actively dividing cells during the first 7 days and slowly proliferating cells from days 7–30. Immunostaining indicated an increase in the number of BrdU^+^ cells in the hMSC-implanted hemisphere within the first day post-implantation when compared to the PBS-injected, contralateral hemisphere (Figure 2 A, E). At 3, 5, 7, and 15 days post-implantation there were still more BrdU^+^ cells in the hMSC-implanted hemisphere than in the contralateral, PBS-injected, control hemisphere (Figure 2 B–D compared to F–H and I compared to L). At 30 days post-implantation, there was a marked decrease in the number of BrdU^+^ cells in both hemispheres (Figure 2 J, M). As a control, dead hMSCs were implanted into the HD striatum. There was no increase in the number of BrdU^+^ cells in the dead hMSC-implanted hemisphere compared to the contralateral PBS-injected hemisphere when analyzed by immunostaining at 7 days post-implantation (Figure 2 K, N). At 5 days post-implantation intact GFP-hMSCs can still be detected; however, confocal microscopy confirmed that these cells were not BrdU^+^ (P-S).

The number of BrdU^+^ cells in each hemisphere was also counted by stereology (Figure 2 O). In the hMSC-implanted hemisphere, there was a progressive increase in BrdU+ cells from 5 to 15 days post-implantation. In the PBS-injected hemisphere, there was a small but not significant increase on day 7 in BrdU+ cells. Most importantly, however, there were significantly more BrdU+ cells in the hMSC-implanted hemisphere than the contralateral, PBS-injected hemisphere at days 5 (p = 0.0421), 7 (p = 0.0293), and 15 days (p<0.001). By day 30, the number of BrdU+ decreased markedly. Implantation of dead hMSCs had no effect on the number of BrdU^+^ cells.

### BrdU^+^ Cells Underwent Neurogenesis

**Figure 3:**
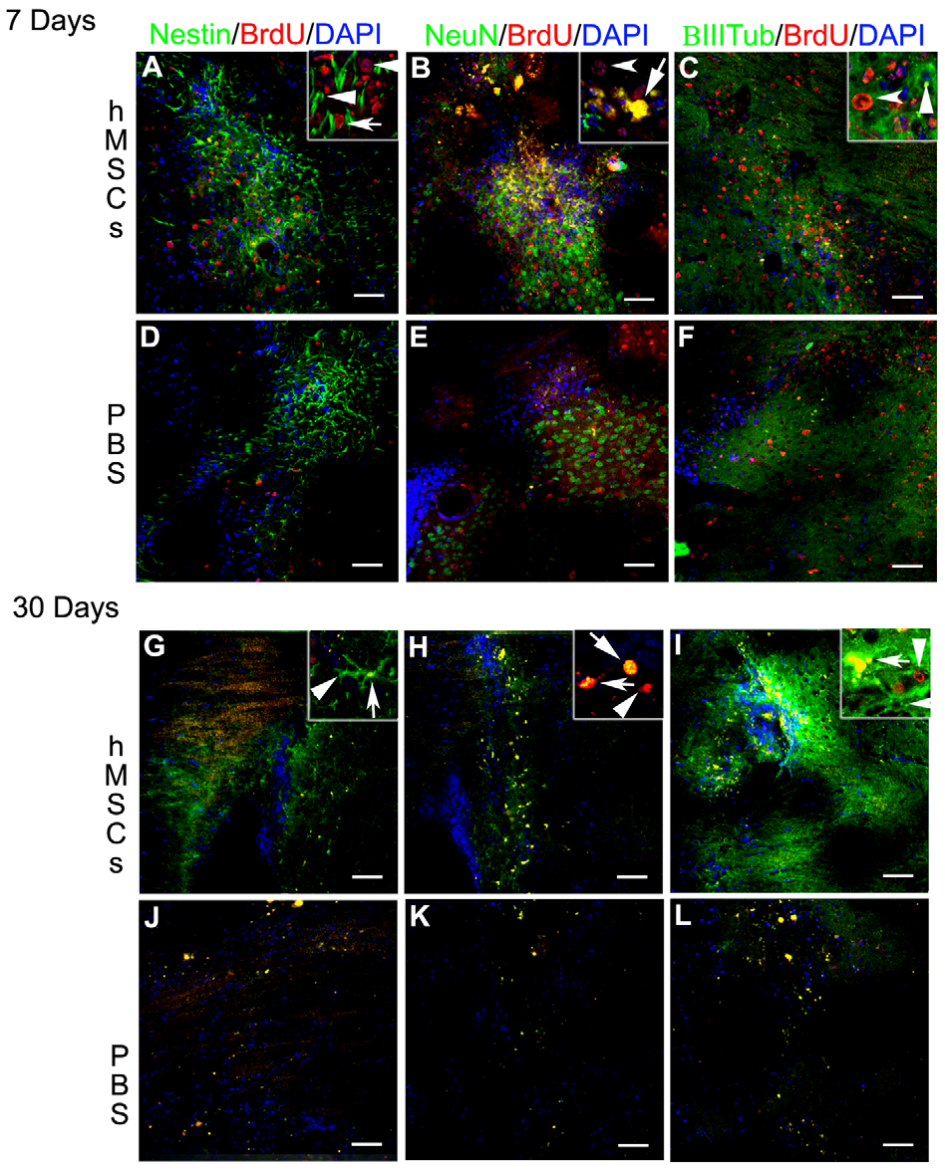
hMSC implantation recruited neuronal cells and induced neurogenesis. There was a slight increase in Nestin^ +^ NPCs to the site of the graft (A) compared to the PBS injection (D) at 7 days post-implantation. Many of these recruited Nestin^+^ NPCs remained proximal to the graft until 30 days (G compared to J). There was also an influx of NeuN^+^ neurons (B) and, to a lesser extent, βIII tubulin^ +^ neurons (C) to the hMSC graft compared to the PBS injection (E,F) at 7 days. At 30 days post-implantation there were still more NeuN^+^ (H) neurons and an overwhelming increase in the number of βIII tubulin^ +^ (I) neurons at the site of the graft compared to the PBS-injected, contralateral hemisphere (K,L). Some BrdU^+^ cells co-labeled with Nestin at 7 days (inset image A) and 30 days (inset image G) post-implantation. A few BrdU^+^ cells were NeuN+ at 7 days post-implantation (inset image B). This number had increased by 30 days post-implantation (inset image H). No BrdU+ cells had matured into βIII tubulin^+^ neurons at 7 days post-transplantation (inset image C). By 30 days, many BrdU^+^ cells did co-express βIII tubulin (inset image I). Squares demarcate location of high magnification inset. Scale bar = 50 µm. Arrows = co-labeled cells, arrowheads = single labeled cells.

To determine whether the BrdU^+^ cells differentiated, sections were co-labeled with markers of differentiation. At 7 days post-implantation, a small number of the BrdU^+^ cells co-labeled with nestin (Figure 3 A, inset images). By 30 days post-implantation, there was an apparent increase in BrdU^+^ cells co-labeled with nestin, but the cells appeared immature with very short processes or without processes (Figure 3 G, inset images). Also, at 7 days post-implantation, many BrdU^+^ cells co-labeled with NeuN (Figure 3 B, inset images). By 30 days post-implantation, BrdU^+^/NeuN^ +^ cells were present but most of the BrdU^+^ cells still did not express NeuN (Figure 3 H, inset images). By 30 days post-implantation, some BrdU^+^ cells expressed βIII tubulin (Figure 3 I, inset images). Therefore, a subset of BrdU^+^ cells differentiated into neurons expressing NeuN and βIII tubulin within the first 30 days post-implantation. Also, confocal imaging of sections demonstrated that a fraction of the BrdU^+^ cells were present as astrocytes and even less as microglia/macrophages (Figure S2). These results suggest that after MSC implantation, BrdU+ cells have the ability to differentiate into neurons and neural progenitor cells within the first 7 days. However, neurogenesis did not appear to be the primary method for repair.

### Grafting hMSCs Appeared to Recruit Pre-Existing Neuronal Cells to the Striatum of HD Mice

In addition to the BrdU^+^ cells that underwent neurogenesis into neural cells, there was also an increase in the number of BrdU negative cells that were nestin^+^, NeuN^+^, and βIII tubulin^+^ in the hMSC-implanted hemisphere compared to the PBS-injected hemisphere. At low magnification at 7 days post-implantation, there was a slight increase in the number of Nestin^+^ cells in the hMSC-implanted hemisphere when compared to the contralateral, PBS-injected hemisphere (Figure 3 A, D). By 30 days post-implantation, there was a considerable increase in the number of nestin^+^ cells in the hMSC-implanted striatum compared to the PBS-injected, contralateral hemisphere (Figure 3 G, J). There was also an increase in the overall number of neurons in the HD striatum after hMSC implantation compared to the PBS-injected control when assessed by NeuN staining at 7 (Figure 3 B, E) and 30 days (Figure 3 H, K). At 7 days post-implantation, there was a slight increase in βIII tubulin+ neurons (Figure 3 C, F). However, by 30 days, the relative number of βIII tubulin+ neurons in the MSC-implanted compared to the PBS-injected hemisphere had increased substantially (Figure 3 I, L). The results indicated either that some neural stem cells whose proliferation was stimulated by the hMSCs were not labeled by BrdU, the hMSCs recruited other progenitor cells the striatum in the HD mice, or hMSC implantation was neuroprotective and increased survival of pre-existing, endogenous striatal neurons. There was no apparent increase in astrocytes, microglia, and macrophages an observation consistent with the fact that the mice were immunosuppressed (Figure S3).

### Grafting hMSCs in the HD Mouse Striatum Increased Neurotrophin Signaling

**Figure 4:**
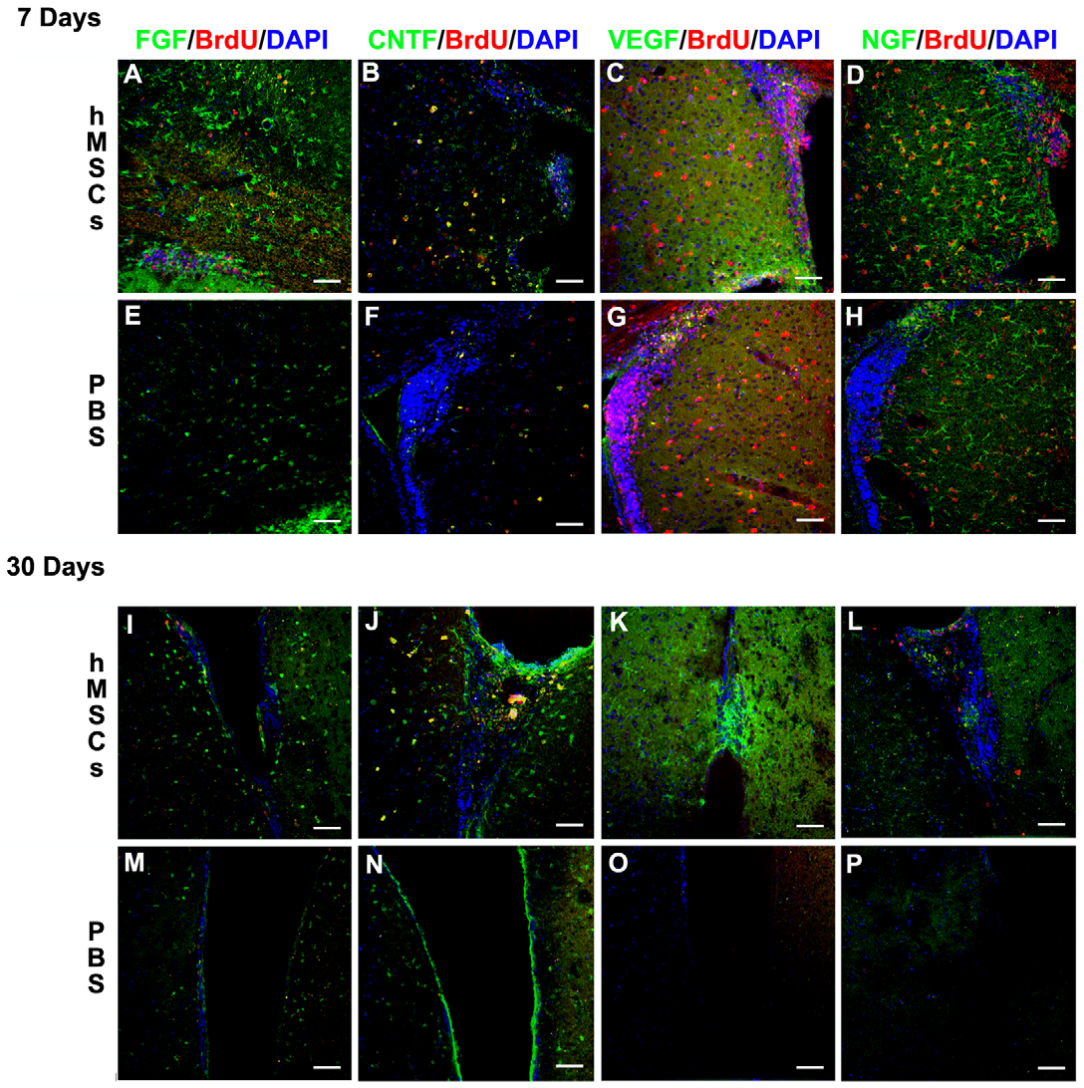
hMSC implantation increased neurotrophic factor signaling. There was a substantial increase in FGF-2 signaling in the hMSC-implanted hemisphere (A, I) compared to the contralateral, PBS-injected hemisphere (E, M) at 7 and 30 days post-implantation. There was no increase in CNTF signaling at 7 days post-implantation (B) compared to the PBS-injected hemisphere (F) although a slight increase was detected by 30 days post-implantation (J compared to N). There was an increase in VEGF signaling in the hMSC-implanted hemisphere at both 7 (C) and 30 (K) days post-implantation compared to the PBS-injected hemisphere (G,O). NGF signaling appeared to be increased similarly in both the hMSC- and the PBS-injected hemispheres 7 days post-implantation (D,H). By 30 days post-implantation, NGF was still slightly increased in the hMSC-implanted hemisphere (L) compared to the contralateral, PBS-injected hemisphere (P). Scale bar = 50 µm.

Assays for FGF-2 signaling indicated a substantial increase in the striatum at 7 days post-implantation when compared to the contralateral, PBS-injected hemisphere (Figure 4 A, E) and a further increase by 30 days post-implantation, especially surrounding the SVZ (Figure 4 I, M). CNTF was not increased at 7 days post-implantation (Figure 4B, F) but there was an increase in the striatum proximal to the SVZ 30 days post-implantation (Figure 4J, N). VEGF signaling was increased at 7 days post-hMSC implantation (Figure 4 C, G), and the increase persisted at 30 days in the areas surrounding the original location of the hMSC graft as well as in the SVZ whereas no VEGF signaling was detected in the contralateral, PBS injected-hemisphere (Figure 4 K, O). NGF signaling was similarly increased in the striatum at 7 days after hMSC implantation (Figure 4D, H) but only slightly increased in the SVZ at 30 days (Figure 4L, P). Therefore, hMSC implantation primarily increased FGF-2 and VEGF signaling at 7 days post-implantation, with increased signaling of FGF-2, CNTF, and VEGF by 30 days post-implantation.

### hMSC Decreased Atrophy of the Striatal Volume in HD Mice

**Figure 5:**
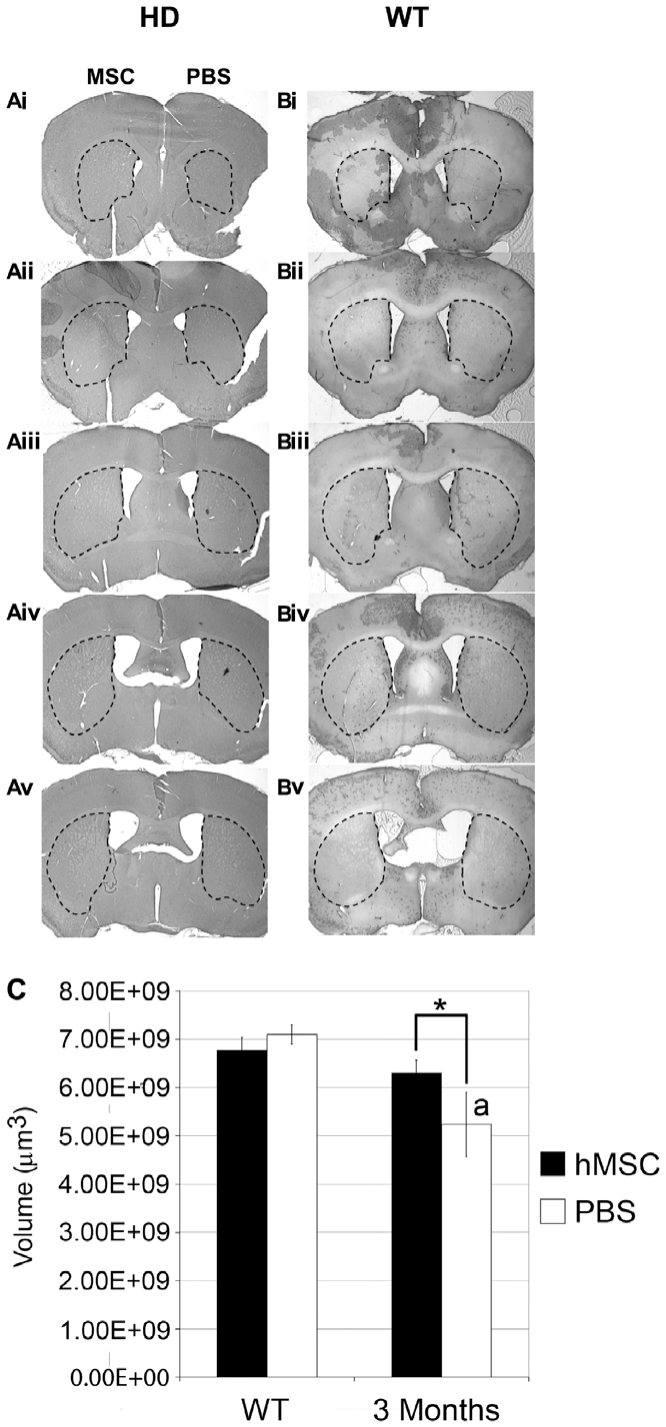
hMSC implantation decreased striatal atrophy in HD mice. Serial sections throughout the striatum were used for striatal volume analysis. Serial sections of the striatum are outlined in both HD mice receiving MSCs or PBS (Ai–Av) and control WT mice (Bi–Bv). There was an apparent increase in striatal volume after MSC implantation at 8 weeks compared to the contralateral, PBS injected hemisphere (Ai–Av). There was no apparent difference in the size of the striatum in the opposite hemispheres in WT mice (Bi–Bv). Scale bar = 1 mm. (C) Stereological analysis at 1 month post-implantation revealed a significant increase in striatal volume after hMSC implantation compared to PBS injection (n = 5−6; *). The PBS-injected striatum was significantly smaller than both the MSC-implanted (a) and PBS WT control striatum (b). There was no difference between the two hemispheres in the WT control group. *, a, and b denote p<0.05 by ANOVA with Holms-Sidak post-hoc analysis.

To assess the effects of hMSCs on HD pathophysiology, hMSCs were implanted into one hemisphere of the striatum and PBS injected into the contralateral striatum in 2-month old HD mice. One month post-implantation, the hMSC-implanted striatum appeared larger than the contralateral, PBS-injected striatum (Figure 5 Ai–Av). This effect was most noticeable in the rostral striatum, close to the implantation site, but was studied throughout 300 µm of the striatum. There were no apparent differences in the contralateral hemispheres of the striatum in wildtype mice (Figure 5 Bi–Bv). Total striatal volume of each experimental and control hemisphere was quantified using stereology. The size of the striatum in wildtype mice was approximately 7×10^9^ µm^3^ and is significantly smaller in HD mice (approximately 5.3×10^9^ µm^3^). The MSC-implanted striatum was significantly larger than the contralateral, PBS-injected hemisphere (approximately 6.3×10^9^ µm^3^, Figure 5C) in 3-month old HD mice. The HD, PBS-injected hemisphere was significantly smaller that the wildtype control hemispheres, but the HD, MSC-implanted hemisphere was not. Therefore, MSC implantation significantly decreased atrophy of the striatum.

## Discussion

This study tested the hypothesis that hMSCs are neurogenic in the striatum of HD mice by increasing the endogenous cell response. Previous reports demonstrated that hMSCs disappeared through necrosis or apoptosis in 5 to 15 days post-implantation into the dentate gyrus of the hippocampus [30]or wildtype adult mice [31], and rat MSCs disappeared at about the same rate after implantation into the striatum of adult rats [32]. A similar rate of disappearance was observed here after implantation of hMSCs into the striatum of adult HD mice. Therefore there was little opportunity for the cells to replace neuronal cells that are lost in HD. Instead, the cells stimulated proliferation and differentiation of endogenous neural cells as indicated by the BrdU labeling experiments. There was also an increase in βIII tubulin^+^ neurons in the striatum that were not BrdU-labeled, an observation suggesting either that there was recruitment of non-stem cells to become neurons, increased neuronal survival or pre-existing neurons, or some neural stem cells escaped labeling with BrdU. The increase in neurogenesis may in itself account for the decreased atrophy of the striatum in the treated HD mice compared to PBS injected controls (Figure 5). However, the hMSCs may also have exerted a neuroprotective effect as was observed in a mouse model for transient cerebral ischemia [31]. Both the increased neurogenesis and any neuroprotective effects were probably exerted through the increase in expression of FGF-2, CNTF, VEGF, and NGF. The increases in FGF-2, CNTF and VEGF persisted for at least 30 days, long after no human cells were detectable. Therefore the transient presence of the hMSCs must have stimulated prolonged expression of neurotrophins by endogenous neural cells in the HD brain. Similar effects were observed after implantation of hMSCs into the dentate gyrus of wildtype, adult mice [30] and of adult mice following transient cerebral ischemia [31]. The results here are consistent with the observation that implantation of autologous bone marrow stromal cells produced improvement in neurological defects in HD mice, experiments in which the fate of the cells was not investigated [33]. The present study is not focused on clinical improvement in treated HD mice but the neurological consequence of an hMSC graft in HD mice; however, a more rigorous study to determine the age of treatment and variability in the phenotype is critical.

The improvement in neurogenesis and striatal volume observed in the HD mice supports previous suggestions that therapy with hMSCs might be effective in CNS diseases such as HD. However, a number of further questions need to be resolved such as appropriate time of the therapy and optimal doses. For example, by simply advancing the surgery by 2 weeks to 6 week-old mice, MSC implantation no longer had a significant effect on striatal volume (data not shown). Administration of MSCs may or may not have long-term benefits, since the endogenous neural cells that undergo neurogenesis contain the mutated gene. However, the hMSCs might reset the time clock for development of the disease phenotype. Observations in models for stroke suggest that intravenous administration of MSCs or conditioned medium from MSCs can be effective [27], [29]. Therefore repeated administration of either the cells or soluble factors produced by the cells may be feasible.

### Conclusions

We have demonstrated that hMSCs affect endogenous cells in the striatum of HD mice by increasing cell proliferation, neuronal differentiation, and neuronal cell recruitment. Furthermore, implantation of hMSCs into the striatum of HD mice prevented striatal atrophy associated with HD. We have documented specific cellular changes in the HD striatum after hMSC implantation. hMSCs are isolated from the bone marrow and, therefore, are readily available for potential therapy or treatment. The results therefore suggested that neural implantation of hMSCs may be of benefit in HD but a number of parameters of dose, treatment schedule, and route of administration need to be optimized.

## Materials and Methods

### Cell Culture

Frozen vials of extensively characterized preparation of hMSCs from normal healthy donors were obtained from the Tulane Center for the Preparation and Distribution of Adult Stem Cells (http://www.som.tulane.edu/gene_therapy/distribute.shtml) under an IRB approved protocol and expanded as described previously [34]. In brief, vials of passage 1 hMSCs (about 1×10^6^ cells) plated in 15 cm-diameter dishes in complete hMSC medium [α-MEM (GIBCO/BRL, Grand Island, NY); 20% pre-screened FBS (Atlanta Biologicals, Norcross, GA); 100 units/ml penicillin; 100 µg/mL streptomycin; and 2 mM L-glutamine (all from GIBCO/BRL)]. After 24 h, the viable adherent cells were washed with phosphate buffered saline (PBS, pH 7.2) and lifted with 0.25% trypsin/1 mM EDTA at 37°C for about 5 min [11] to recover viable cells. The cells were replated at 50 to100 viable cells per cm^2^ in hMSC medium, incubated with a change of medium every 3 or 4 days for 7 days until reaching about 70% confluent, and lifted with trypsin/EDTA. The epitope profile of cells was: Negative for hematopoietic markers (CD34, CD36, CD117 and CD45), and positive for CD29 (95%), CD44 (>93%), CD49c (99%), CD49f (>70%), CD59 (>99%), CD90 (>99%), CD105 (>99%) and CD166 (>99%). For transplantation, the cells were washed with PBS, centrifuged, and re-suspended in PBS at 25,000 cells/µL. To create Green Fluorescent Protein (GFP)-hMSCs, GFP lentivirus with an ubiquitin promoter [35] and polybrene were added to the culture media for 24 hours. At least 95% of the hMSCs were GFP+. GFP-hMSCs were re-plated post-surgery to confirm viability (Figure S4).

### Stereotactic Surgery

All protocols involving animal care and handling were approved by Emory University's Institutional Animal Care and Use Committee. N171-82Q (Jackson Laboratories, Bar Harbor, Maine) 8-week old mice were used for stereotactic surgery. They were immunosuppressed with 10 mg/kg cyclosporine A administration subcutaneously daily for 3 days prior to and 15 days after surgery. During Cyclosporine A treatment, mice were housed in sterile cages with sterile food and water. After pre-treatment with 0.04 mg/kg Atropine, they were anesthetized using 90.0 mg/mL ketamine and 9.1 mg/mL xylazine intramuscularly. The cranium was exposed and 4 µL of hMSC suspension in PBS (25,000 cells/µL) were infused at a rate of 0.5 µL/min at: +0.74 anterior/posterior, +/−1.5 medial/lateral, −2.5 dorsal/ventral relative to Bregma [36] into the right striatum. The dwell time was 5 min. The same volume of PBS was injected at the same rate in the left hemisphere. The wound was closed using Vetbond (Fisher Scientific, Inc.) and mice were administered 2.5 mg/kg Flunixin for recovery. For labeling of stem cells, 50 mg/kg bromodeoxyuridine (BrdU) neutralized in PBS with 0.007N NaOH was administered intraperitoneally at the time of surgery and at 12 hour intervals for 7 days.

### Perfusion and Brain Tissue Processing

All protocols involving animal care and handling were approved by Emory University's Institutional Animal Care and Use Committee. Mice were euthanized with avertin intraperitoneally, they were perfused through the left ventricle with ice-cold PBS followed by 4% paraformaldehyde. The brains removed and post-fixed in 4% paraformaldehyde overnight at 4°C, transferred to fresh 30% sucrose for 2 days and then frozen with 2-methylbutane. Sections were cut at 40–50 µm using a cryostat.

### Immunohistochemisty

Sections requiring detection of bromodeoxyuridine (BrdU) were first pretreated with 30 min in 2N Hydrochloric acid at 37°C followed by 15 min in borate buffer at room temperature. Sections were blocked using 5% serum in Triton-X 100. Incubation in the primary antibody was overnight at 4°C and in the secondary antibody was 1 hour at room temperature. Primary antibodies used include: BrdU (Abcam Inc, Cambridge, ME), Nestin (Chemicon International, Temecula, CA), neuronal nuclei (Chemicon International), βIII Tubulin (Chemicon International), glial fibrillary acidic protein (Chemicon International), Iba1 (Wako Pure Chemical Industries, Ltd, Richmond, VA), ciliary neurotrophic factor (Santa Cruz Biotechnology, Inc.), fibroblast growth factor-2 (Santa Cruz Biotechnology, Inc.), nerve growth factor (Santa Cruz Biotechnology, Inc.), vascular endothelial growth factor (Chemicon International). Sections were mounted and coverslipped using Vectashield (Vector Laboratories, Inc, Burlingame, CA).

### Hematoxylin and Eosin Stain

Sections were mounted and allowed to adhere to the slide overnight. Sections were incubated in Hematoxylin for 10 seconds and Eosin for 5 seconds. Sections were rehydrated with water and mounted with aqueous media.

### Microscopy

All confocal images were taken using the Zeiss LSM 510 Meta Confocal and corresponding software.

### Stereology

All sections to be used for stereology were sectioned at 50 µm and examined with a spinning disc confocal microscope. Stereo Investigator (Microbrightfield Bioscience, Williston, VT) was used to count cells. Cells were counted in every sixth section. Each mouse for stereological assessment received an hMSC graft in the right hemisphere and a PBS injection in the left hemisphere. The total number of GFP+ was counted in every sixth section. No ratio was needed for the MSC graft volume estimates because the entire size of the graft was calculated. The entire striatum was outlined in every sixth section and randomly sampled to count for BrdU+ cells. For analysis, both the total number of BrdU+ cells in each hemisphere as well as the ratio of BrdU+ cells in the hMSC-implanted hemisphere to those in the PBS-injected hemisphere was calculated to reduce potential differences in data collection between subjects. To quantify the volume of the striatum, the area of the entire striatum was measured in every third section. Based on the area in each section, the number of sections, plus the thickness of each section, the total volume per hemisphere was calculated. The ratio of the volume in the MSC-implanted hemisphere to that of the PBS-injected hemisphere was also determined.

### Statistics

A one-way ANOVA was used to determine if any of the groups were significantly different from the rest. A One-way RM ANOVA was also used to determine any differences within each group. The post-hoc test used was the Holms-Sidak test.

## Supporting Information

Figure S1 Confocal microscopy of GFP-hMSCs *in vivo*. High magnification confocal microscopy confirmed GFP-hMSCs did not divide *in vivo*. Intact GFP-hMSCs did not label (arrows) with BrdU at 1 day (A–D), 3 days (E–H), or 5 days (I–L). As hMSCs died, some GFP-hMSC remnants were phagocytosed by surrounding cells (arrowheads), including BrdU^+^ cells at 5 days (I–L), 7 days (M–P), 15 days (Q–T), and 30 days (U–X). Scale bar = 40 µm.
(8.78 MB TIF)

Figure S2 BrdU^+^ cells underwent gliogenesis. (A) A few BrdU^+^ cells differentiated into astrocytes by 7 days post-implantation. (B) By 30 days, many BrdU^+^ cells underwent gliogenesis into mature astrocytes with long, elaborate processes. A few BrdU^+^ cells had differentiated into mature microglia/macrophages at 7 (C) and 30 days (D) post-implantation. Arrows = co-label examples. Arrowheads = single-label examples. Scale bar = 40 µm.
(10.19 MB TIF)

Figure S3 Grafting hMSCs did not recruit pre-existing, endogenous glial cells. There was no difference in the number of GFAP^+^ astrocytes in the hMSC-implanted hemisphere (A, C) compared to the PBS-injected hemisphere (E, G) at 7 or 30 days post-implantation. There was also no difference in the number of Iba1^+^ microglia/macrophages at the site of the hMSC graft (B, D) compared to the PBS injection (F, H) at 7 or 30 days post-implantation. Scale bar = 50 µm.
(6.21 MB TIF)

Figure S4 Morphology of hMSCs. hMSCs were plated at 100 cells/cm2 and grown to 70% confluency before harvesting for surgery (A). Cells were infected with GFP during their post-thaw, recovery phase. DIC images reveal the typical morphology of MSCs and GFP fluorescence microscopy showed almost 100% expression. Cells were re-plated immediately following surgery and still proved to be viable (B).
(3.41 MB TIF)
