# Supplementary figures and images for: Human Multipotent Stromal Cells (MSCs) Increase Neurogenesis and Decrease Atrophy of the Striatum in a Transgenic Mouse Model for Huntington's Disease
Source: PLoS One. 2010 Feb 22;5(2):e9347. doi: 10.1371/journal.pone.0009347 (PMC2825266; doi:10.1371/journal.pone.0009347)

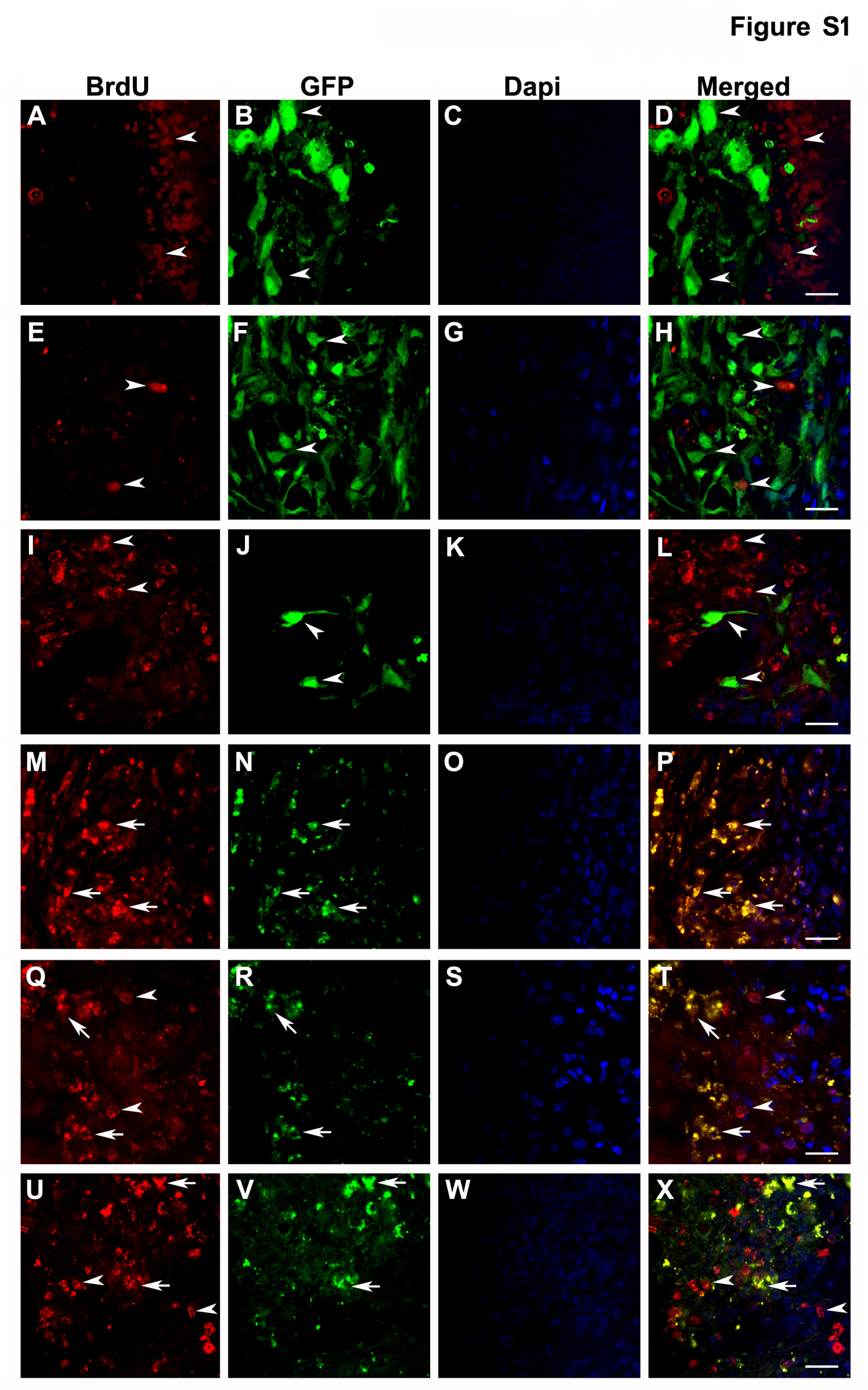

Supplement: Figure S1 — Confocal microscopy of GFP-hMSCs in vivo. High magnification confocal microscopy confirmed GFP-hMSCs did not divide in vivo. Intact GFP-hMSCs did not label (arrows) with BrdU at 1 day (A–D), 3 days (E–H), or 5 days (I–L). As hMSCs died, some GFP-hMSC remnants were phagocytosed by surrounding cells (arrowheads), including BrdU+ cells at 5 days (I–L), 7 days (M–P), 15 days (Q–T), and 30 days (U–X). Scale bar = 40 µm. (8.78 MB TIF) [file pone.0009347.s001.tif]

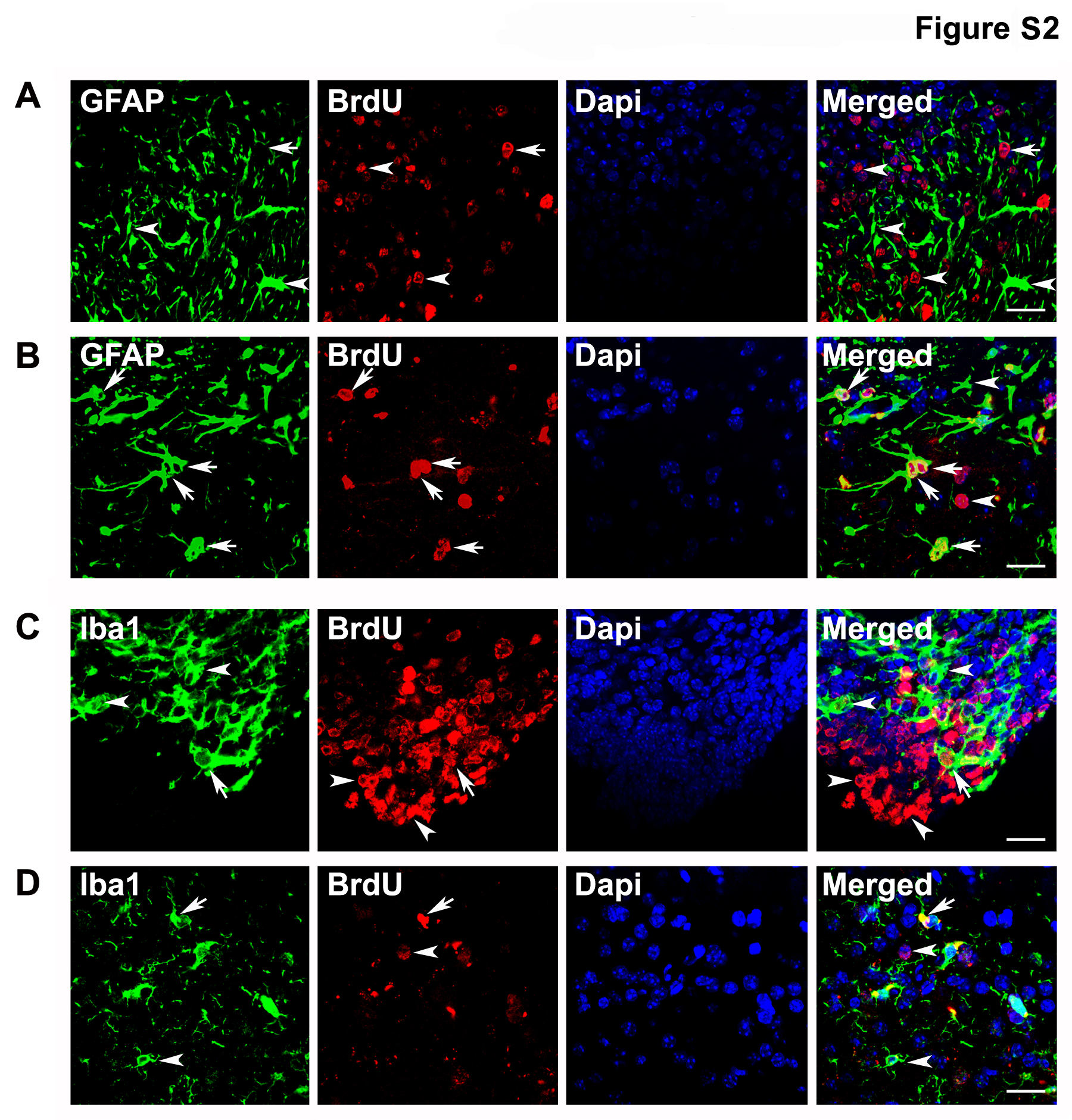

Supplement: Figure S2 — BrdU+ cells underwent gliogenesis. (A) A few BrdU+ cells differentiated into astrocytes by 7 days post-implantation. (B) By 30 days, many BrdU+ cells underwent gliogenesis into mature astrocytes with long, elaborate processes. A few BrdU+ cells had differentiated into mature microglia/macrophages at 7 (C) and 30 days (D) post-implantation. Arrows = co-label examples. Arrowheads = single-label examples. Scale bar = 40 µm. (10.19 MB TIF) [file pone.0009347.s002.tif]

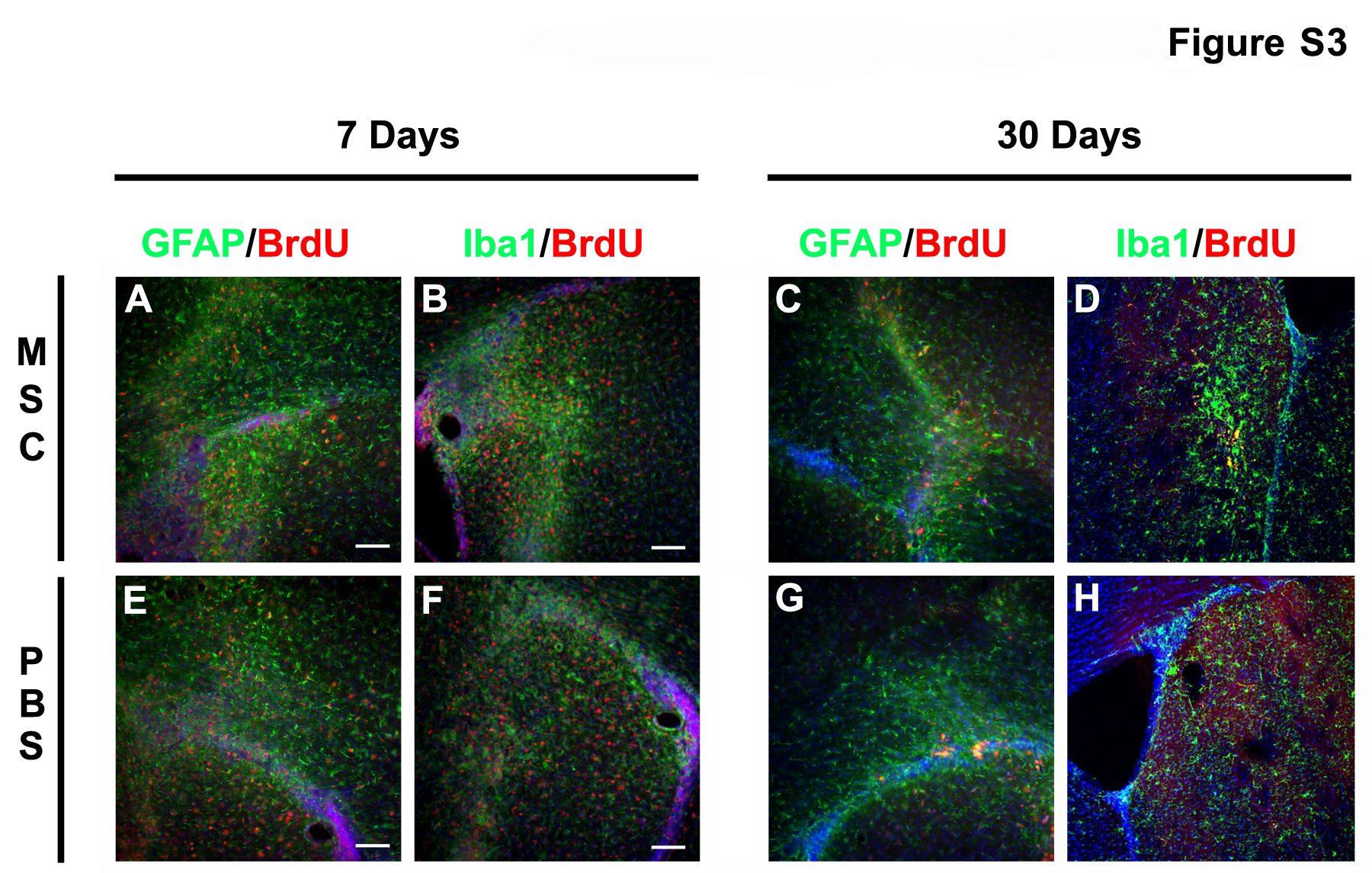

Supplement: Figure S3 — Grafting hMSCs did not recruit pre-existing, endogenous glial cells. There was no difference in the number of GFAP+ astrocytes in the hMSC-implanted hemisphere (A, C) compared to the PBS-injected hemisphere (E, G) at 7 or 30 days post-implantation. There was also no difference in the number of Iba1+ microglia/macrophages at the site of the hMSC graft (B, D) compared to the PBS injection (F, H) at 7 or 30 days post-implantation. Scale bar = 50 µm. (6.21 MB TIF) [file pone.0009347.s003.tif]

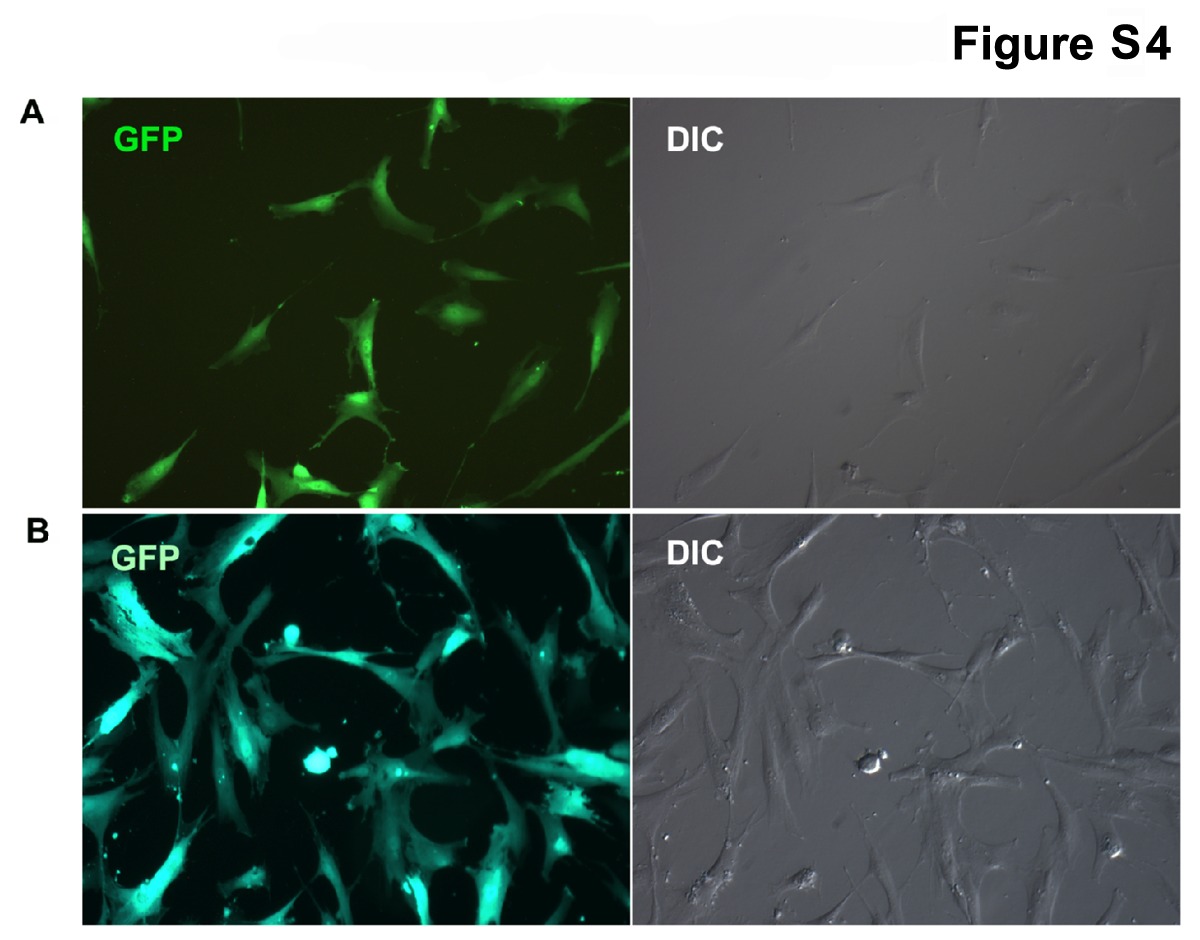

Supplement: Figure S4 — Morphology of hMSCs. hMSCs were plated at 100 cells/cm2 and grown to 70% confluency before harvesting for surgery (A). Cells were infected with GFP during their post-thaw, recovery phase. DIC images reveal the typical morphology of MSCs and GFP fluorescence microscopy showed almost 100% expression. Cells were re-plated immediately following surgery and still proved to be viable (B). (3.41 MB TIF) [file pone.0009347.s004.tif]
